# Living and Regenerative Material Encapsulating Self-Assembled *Shewanella oneidensis*-CdS Hybrids for Photocatalytic Biodegradation of Organic Dyes

**DOI:** 10.3390/microorganisms10122501

**Published:** 2022-12-16

**Authors:** Mingyue Tao, Chenyang Jin, Hongfei Lu, Kai Jin, Lin Yu, Jinliang Liu, Jing Zhang, Xiaohui Zhu, Yihan Wu

**Affiliations:** 1 Department of Chemical and Environmental Engineering, Shanghai University, Shanghai 200433, China; 2 Medical School, Shanghai University, Shanghai 200433, China

**Keywords:** nano-bacteria hybrid, hydrogel, biodegradation, *Shewanella oneidensis*, living material

## Abstract

Reductive biodegradation by microorganisms has been widely explored for detoxifying recalcitrant contaminants; however, the biodegradation capacity of microbes is limited by the energy level of the released electrons. Here, we developed a method to self-assemble *Shewanella oneidensis*-CdS nanoparticle hybrids with significantly improved reductive biodegradation capacity and constructed a living material by encapsulating the hybrids in hydrogels. The material confines the nano-bacteria hybrids and protects them from environmental stress, thus improving their recyclability and long-term stability (degradation capacity unhindered after 4 weeks). The developed living materials exhibited efficient photocatalytic biodegradation of various organic dyes including azo and nitroso dyes. This study highlights the feasibility and benefits of constructing self-assembled nano-bacteria hybrids for bioremediation and sets the stage for the development of novel living materials from nano-bacteria hybrids.

## 1. Introduction

Microbial biodegradation has been extensively investigated as a simple, cost-effective, and environment-friendly strategy for detoxifying pollutants. Microbes have been demonstrated to degrade a variety of chemicals, such as small-molecule organic dyes and synthetic plastics [1,2]. Under aerobic conditions, organic contaminants can be oxidized by microorganisms and utilized as carbon or energy sources through aerobic respiration [3]. In addition to oxidization, microbial reductive biodegradation has been explored to biotransform recalcitrant pollutants such as halogenated chemicals and azo dyes [4]. During reductive biodegradation, microbes can reduce contaminants through their reductases. For instance, azo-reductases in bacteria and fungi have been characterized and recognized as enzymes contributing to the decolorization of azo dyes [5].

Because enzymes have substrate specificity, the application of enzyme-based biodegradation is limited to certain compounds with specific functional groups. A more general approach can be developed by harnessing the reductive degradation capacity of electrochemically active bacteria (EAB). These EAB are capable of respiring using extracellular electron acceptors through extracellular electron transfer. Chemicals such as azo dyes can capture electrons released from EAB and can be reduced without catalysis by azo reductases [6]. However, the reductive biodegradation capacity of bacterial cells is limited by the energy levels of the electrons released from the EAB. Strategies to aid electron transfer or enhance the energy level of electrons from the EAB can improve the reductive degradation performance [7].

**Figure 1:**
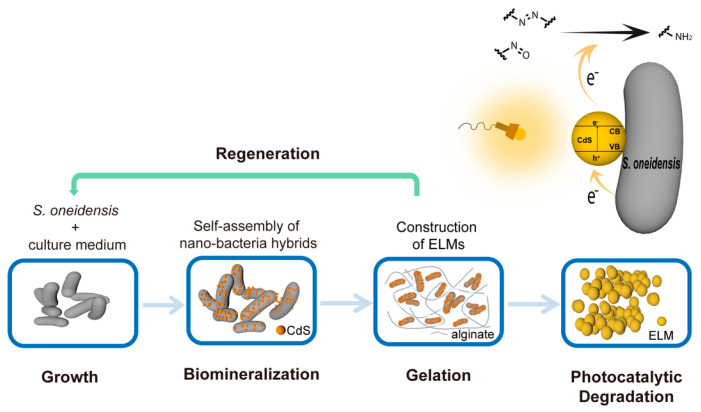
Schematic showing the workflow to construct engineered living materials (ELMs) with encapsulated nano-bacteria hybrids. The CdS nanoparticles biomineralized by *Shewanella oneidensis* are deposited on the surface of cells to self-assemble nano-bacteria hybrids. The hybrids are encapsulated in an alginate hydrogel containing nutrients to form ELMs. Nano-bacteria hybrids in the ELMs can remove contaminants via photocatalytic reductive degradation.

Attempts have been made to engineer bacterial cells by constructing nano-bio hybrids. For example, nano-bacteria hybrids constructed from non-photosynthetic bacteria can harvest solar energy and produce acetic acids from CO_2_. Nano-bio hybrids have recently been applied in diverse fields including catalysis, energy storage, and electronics. Here, we report an approach for the *in situ* self-assembly of *S. oneidensis*-CdS nanoparticle hybrids under pseudo anaerobic conditions, which can be directly employed for the photocatalytic degradation of organic dyes (Figure 1). *Shewanella oneidensis* is a Gram-negative facultative anaerobe with a sophisticated respiratory network that uses diverse electron acceptors [8,9]. Owing to its prominent capability for extracellular electron generation and transfer, *S. oneidensis* has been explored for a wide range of applications including biodegradation [10,11,12,13,14,15,16]. The biodegradation performance of the hybrids was significantly improved compared to that of *S. oneidensis* without engineering. The application of nano-bio hybrids is primarily limited by the fragility and susceptibility of hybrid cells [17]. To address the challenges, we constructed an engineered living material (ELM) [18,19] based on the *S. oneidensis*-CdS hybrids (Figure 1). The effectiveness of the ELMs for the photocatalytic degradation of trypan blue (containing azo bonds) and naphthol green B (NGB; containing nitroso groups) was demonstrated. The material was self-sufficient with nutrients to support encapsulated live hybrid bacterial cells. These cells could survive the construction and storage processes and maintain their ability to photocatalytically degrade organic pollutants. This functional living material improved the stability of the nano-bacteria hybrids and prevented their escape. Outside ELMs, nano-bacteria hybrids cannot survive beyond 3 days, whereas inside ELMs, their viability and photocatalytic degradation capacity remained unhindered after 4 weeks. The ELMs also possessed regenerative abilities; that is, successive regeneration of new ELMs could be performed using one parent ELM containing nano-bacteria hybrids as the seed. Our study highlights the feasibility and benefits of constructing self-assembled nano-bacteria hybrids for bioremediation and sets the stage for the development of novel living materials from nano-bacteria hybrids.

## 2. Materials and Methods

### 2.1. Materials

*Shewanella oneidensis* MR-1 (JCM31522) was obtained from the Typical Culture Conservation Center of China. Luria Bertani (LB) broth and phosphate-buffered saline (PBS) powder were purchased from Solarbio. Sodium alginate, lactic acid, urea, piperazine-1,4-bis (2-ethanesulfonic acid) monosodium salt (PIPES), cadmium chloride, and sodium thiosulfate were purchased from Shanghai Titan Scientific. Modified Wolfe’s mineral solution (1.5 g/L nitrilotriacetic acid, 3 g/L MgSO_4_•7H_2_O, 0.5 g/L MnSO_4_•H_2_O, 1 g/L NaCl, 0.1 g/L FeSO_4_•7H_2_O, 0.1 g/L CoSO_4_•7H_2_O, 0.1 g/L CaCl_2_•2H_2_O, 0.1 g/L ZnSO_4_•7H_2_O, 0.01 g/L AlK (SO_4_)_2_, 0.01 g/L H_3_BO_3_, 0.01 g/L Na_2_MoO_4_•2H_2_O, 0.01 g/L CuSO_4_•5H_2_O, and 0.01 g/L NiCl_2_•6H_2_O, pH = 7) and Wolfe’s vitamin solution (20 mg/L biotin, 20 mg/L folic acid, 100 mg/L pyridoxine hydrochloride, 50 mg/L thiamine hydrochloride, 50 mg/L riboflavin, 50 mg/L nicotinic acid, 50 mg/L calcium D- (+)-pantothenate, 1 mg/L vitamin B12, 50 mg/L p-aminobenzoic acid and 50 mg/L thioctic acid) were purchased from Biofeng. Lactic acid was filtered through a 0.22 μm filter membrane before use.

### 2.2. Methods

#### 2.2.1. Self-Assembly of Nano-Bacteria Hybrids

A single colony of *S. oneidensis* grown on LB agar was used to inoculate 5 mL of LB medium in a 14 mL culture tube. The culture was incubated at 30 °C and 250 rpm for 12 h. A fresh LB medium solution (5 mL) in a 14 mL culture tube was inoculated with 50 μL of the 12-h culture and cultured at 30 °C and 250 rpm. After 24-h incubation, the bacterial cells were collected by centrifugation at 4500× *g* for 10 min. The cell pellet was washed three times with 10 mL of sterile PBS solution and then 10 mL of mineral medium (0.3 g NaOH, 1.5 g NH_4_CI, 0.1 g KCI, 5.85 g NaCl, 15.12 g PIPES, 1 mL Wolfe’s vitamin solution, 1 mL modified Wolfe’s mineral solution, and 9 mM lactic acid dissolved in 1 L of ultrapure water). After washing, the cell pellet was resuspended in a mineral medium to obtain a solution with a cell density of OD_600 nm_ = 0.5. Na_2_S_2_O_3_ (final concentration, 2 mM) and lactic acid (final concentration, 9 mM) were added to the cell suspension. Capped culture tubes filled with the as-prepared cell suspension (microaerobic conditions) were incubated at 30 °C for 24 h. CdCl_2_ was added at a final concentration of 1 mM and incubated at 30 °C for an additional 48 h.

#### 2.2.2. TEM/EDS/SAED Analysis of Nano-Bacteria Hybrids

After biosynthesis, the nano-bacteria hybrids were collected from 0.01 mL of solution and washed three times with PBS. A small drop of the resulting diluent was placed on a carbon grid and heated to dehydrate. The as-prepared sample was analyzed using a HITACHI-HT7820 TEM and JEM-2010F HRTEM.

#### 2.2.3. Characterization of CdS Isolated from Nano-Bacteria Hybrids

The nano-bacteria hybrids from 420 mL of the biosynthesis culture were washed with water and resuspended in 20 mL of water. The mixture was then processed using an ultrasonic homogenizer at a power density of 350 W for 2 h. After homogenization, the pellet was collected and heated at 30 °C for 1 h in a solution containing 50 mM Tris-HCl, 50 mM NaCl, and 8 M urea (50 mL; pH 7.5). The precipitate was spun down from the resulting mixture and heated at 30 °C for 1 h in a solution containing 50 mM Tris-HCl, 50 mM NaCl, and 0.2% Tween-20 (50 mL, pH 7.5). The collected precipitate was washed three times each with PBS (50 mL), ethanol (20 mL), and cyclohexane (20 mL). After thorough washing, the isolated CdS nanoparticles were freeze-dried for 12 h. The fluorescence spectrum (excitation wavelength of 350 nm) was measured on a spectrofluorometer (FS5) using a suspension of isolated CdS in a mineral solution.

#### 2.2.4. Degradation of Trypan Blue or NGB by Nano-Bacteria Hybrids

Nano-bacteria hybrids from 56 mL of biosynthesis culture were washed three times with mineral medium (20 mL) and added to a mineral medium solution (50 mL) containing 20 mg/L trypan blue or 100 mg/L NGB. The resulting mixture was irradiated in a capped 50 mL falcon tube with a tungsten filament lamp (60 W) at a power density of 12.9 mW/cm^2^. The control group samples (isolated CdS or *S. oneidensis* cells) were washed and resuspended using the same procedure. The non-irradiated control groups were wrapped in tin foil. The concentrations of trypan blue and NGB over time were determined based on absorbance at 583 nm and 714 nm, respectively.

#### 2.2.5. Preparation of ELMs

Nano-bacteria hybrids collected from 56 mL of biosynthesis culture were washed three times with LB (10 mL) and then resuspended in 5 mL of LB. The resuspension was mixed with 2 mL sterilized sodium alginate solution (0.1 g/mL). The as-prepared mixture was added dropwise (1 drop every three seconds) to a CaCl_2_ (0.1 M) solution using a syringe. The hydrogel spheres encapsulating the nano-bacteria hybrids were washed three times with a PBS solution (60 mL). For the control groups containing unmodified *S. oneidensis* cells, 56 mL of cell culture without CdCl_2_ was used. For the control groups containing only isolated CdS nanoparticles, CdS nanoparticles were isolated from 56 mL of nano-bacteria hybrid solution following the procedure described above and resuspended in 5 mL of LB to be mixed with the sodium alginate solution.

#### 2.2.6. Degradation of Trypan Blue or NGB with ELMs

ELM spheres with encapsulated nano-bacteria hybrids were added to 50 mL of a mineral medium solution containing 20 mg/L trypan blue or 100 mg/L NGB. The samples were irradiated at room temperature with a tungsten filament lamp (60 W, 250–3000 nm) at a power density of 12.9 mW/cm^2^ in capped 50 mL falcon tubes. The non-irradiated control groups were wrapped in tin foil. The concentrations of trypan blue and NGB over time were determined based on absorbance at 583 nm and 714 nm, respectively. In addition to the mineral medium solution, the same degradation procedure was performed in PBS and water.

#### 2.2.7. Recycling and Recharging of ELMs

After degradation, ELM spheres were collected by filtration, washed three times with 20 mL PBS, and then incubated in LB (20 mL) at 30 °C for 24 h. After incubation, the spheres were collected by filtration and washed three times with 20 mL mineral medium solution. The degradation capacity of recycled ELM spheres was assayed according to the procedure described above.

#### 2.2.8. Assessment of Cell Viability in ELMs

The ELMs were incubated in 20 mg/L trypan blue aqueous solution for 24 h with or without irradiation. The ELMs incubated in water without trypan blue were used as control groups. After 24 h of incubation, 10 hydrogel spheres were washed three times with 60 mL of PBS and subsequently incubated with 2 mL of EDTA solution (0.1 M EDTA, 0.2 M K_2_HPO_4_, pH 6.9). A dilution of the resulting solution was plated on an LB agar plate and incubated at 30 °C for 24 h to count the colonies. The resulting solution was subjected to fluorescence microscopy, flow cytometry, and TEM analyses. The samples were stained with a mixture of SYTO 9 and PI for 25 min and analyzed using a confocal laser scanning microscope (OLYMPUS FV3000) and a flow cytometer (CytoFLEX). For TEM analysis, 10 µL of the sample was dropped onto a carbon grid and dried. After recycling, the same analyses were performed on the recovered ELMs.

#### 2.2.9. Biosafety Assessment of ELMs

After 12 h of degradation, the ELMs were removed from the solution via filtration. Then, 0.1 mL of the residual solution was plated on an LB agar plate and incubated at 30 °C. After more than 24 h, the plates were inspected to determine whether any colonies had formed. The same procedure was performed for the samples after a 36-h degradation process.

#### 2.2.10. Stability Assessment of ELMs

The ELM spheres were stored at 4 °C, −20 °C, and −80 °C. The degradation capacity of the samples was assayed after 14 and 28 days to evaluate the stability of the ELMs under different storage conditions.

#### 2.2.11. Regeneration of ELMs

After 24 h of degradation, the ELM spheres were crushed after washing three times with water (10 mL). The crushed spheres were incubated in 5 mL of LB medium at 30 °C and 250 rpm in a 14 mL culture tube. After 12 h, 10 μL of the suspension was plated on an LB agar plate and incubated at 30 °C. After 24-h incubation, a colony was picked from the plate and inoculated into 5 mL of LB medium. After incubation at 30 °C and 250 rpm for 24 h, the resulting cell culture was used for the biosynthesis of nano-bacteria hybrids and regeneration of ELMs. This procedure was repeated for 10 generations.

## 3. Results and Discussion

### 3.1. Self-Assembly and Characterization of Nano-Bacteria Hybrids

As *S. oneidensis* is a facultative anaerobic bacterium, it can consume oxygen via aerobic respiration. We speculated that *S. oneidensis* could deplete dissolved oxygen molecules to generate a pseudo anaerobic environment [20] and use Na_2_S_2_O_3_ as the electron acceptor producing S^2−^ to form CdS with Cd^2+^. Thus, a strict anoxic culturing process is not required. *Shewanella oneidensis* was cultured in capped culture vials supplemented with Na_2_S_2_O_3_ as the electron acceptor. The headspace volume was maintained at 5% of the vials and was not exchanged to eliminate oxygen. Upon addition of CdCl_2_ to the *S. oneidensis* culture, the culture solution gradually turned yellow, indicating CdS nanocrystal formation (Figure S1).

**Figure 2:**
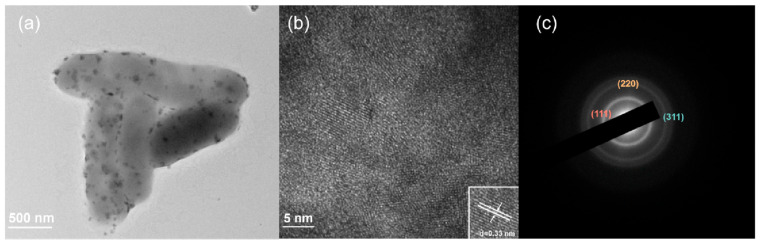
Characterization of the CdS-*Shewanella oneidensis* hybrids self-assembled under pseudo anaerobic conditions: (**a**) TEM image of nano-bacteria hybrids showing self-biomineralized CdS nanocrystals deposited on the cell surface; (**b**) Electron diffraction pattern of the CdS nanocrystals in nano-bacteria hybrids from HRTEM results. The d-spacing of the CdS nanoparticles is 3.3 Å which aligns with the (111) plane of face-centered cubic CdS; (**c**) SAED analysis of the CdS nanocrystals in nano-bacteria hybrids. The indices are assigned to the diffraction rings in accordance with the face-centered cubic lattice of CdS.

Self-biomineralized CdS nanoparticles were deposited on the surface of the *S. oneidensis* cells (Figure 2a). The formation of such nano-bacteria hybrids did not significantly alter the morphology of *S. oneidensis* cells, suggesting that the integrity of cell membranes was preserved, which is critical for electron generation and transfer. To determine the chemical composition and morphology of the biosynthesized CdS nanoparticles, they were isolated from the nano-bacteria hybrids. The SEM-EDS results demonstrated that the ratio of Cd to S atoms in the crystals was nearly 1:1, suggesting that the chemical composition was close to that of stoichiometric CdS (Figure S2). According to the high-resolution TEM results, the d-spacing of the isolated CdS nanoparticles was 3.3 Å which aligned with the (111) plane [21] of cubic CdS (Figure 2b). The electron diffraction ring pattern agreed with that of CdS in the face-centered cubic structure (Figure 2c) [22]. The fluorescence spectrum excited at 350 nm showed an emission peak at 450 nm, which is comparable to that of the chemically synthesized CdS (Figure S3) [23].

Collectively, these results unambiguously suggest that *S. oneidensis*-CdS nano-bacteria hybrids were constructed by supplementing the microaerobic *S. oneidensis* cultures with Na_2_S_2_O_3_ and CdCl_2_. The CdS nanoparticles biosynthesized and deposited on *S. oneidensis* cells had face-centered cubic structures.

### 3.2. Photocatalytic Degradation of Azo Dyes by Nano-Bacteria Hybrids

The semiconductor CdS nanoparticles in the nano-bacteria hybrids can accept electrons from *S. oneidensis* cells. By absorbing light, CdS nanoparticles can excite the accepted electrons to a higher energy level for better reductive degradation. To determine whether the CdS nanoparticles can promote reductive degradation by *S. oneidensis*, the degradation performance of nano-bacteria hybrids for trypan blue (organic dye with azo groups) was compared to that of *S. oneidensis* without engineering. Because oxygen competes with contaminants as electron acceptors, reductive biodegradation is commonly performed under rigorous anaerobic conditions to prevent inhibition by oxygen [24]. Because *S. oneidensis* is a facultative anaerobe capable of both anaerobic and aerobic respiration [25,26], we speculated that the nano-bacteria hybrids could perform reductive biodegradation under microaerobic conditions as the oxygen would be consumed. Compared to other microbes explored for bioremediation (e.g., *Geobacter sulfurreducens*), the system is more robust and easier to handle, without the need for rigorous anaerobic operation.

**Figure 3:**
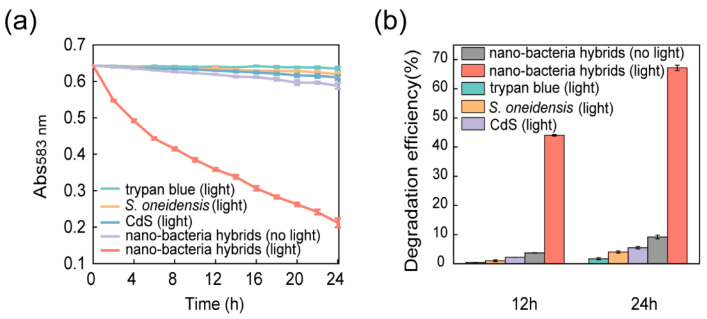
Photocatalytic degradation of trypan blue by nano-bacteria hybrids: (**a**) Change in concentration of trypan blue after treatment with nano-bacteria hybrids and controls. Nano-bacteria hybrids were incubated in a mineral medium solution containing 20 mg/L trypan blue and irradiated by a tungsten filament lamp. The absorbance of the reaction mixture at 583 nm was used to determine the change in concentration of trypan blue. Control groups included trypan blue only (with light), *Shewanella oneidensis* only (with light), CdS only (with light), and nano-bacteria hybrids (without light). The photocatalytic degradation performance of nano-bacteria hybrids was substantially improved compared to that of wild-type *S. oneidensis* cells; (**b**) The concentration of trypan blue after 12 h and 24 h of treatment was compared with the initial concentration to assess trypan blue degradation efficiency. The concentration was determined by comparing it to a trypan blue calibration curve generated based on Abs_583 nm_. The photocatalytic degradation efficiency of nano-bacteria hybrids was 33 times better than that of wild-type *S. oneidensis* cells. The data are presented as mean ± s.d (*n* = 2).

The photocatalytic degradation of azo dyes by the nano-bacteria hybrids was assayed under microaerobic conditions. Nano-bacteria hybrid and wild-type *S. oneidensis* cells with the same number of colony-forming units were incubated in a mineral medium solution containing 20 mg/L trypan blue (simulating wastewater containing dyes and heavy metals). The reduction in the azo bond to the amine group in trypan blue results in a decrease in the characteristic absorption at 583 nm. The absorbance of the reaction mixture at 583 nm was therefore used to determine the degradation efficiency of each sample. The reaction mixture was sampled and measured every 2 h (Figure 3a). The degradation efficiency of the nano-bacteria hybrids was 33-fold higher than that of *S. oneidensis* cells (Figure 3b, Figure S4). After 12 h of treatment, the sample treated with nano-bacteria hybrids under light irradiation turned almost colorless, whereas the other control samples remained blue.

Without light irradiation, the degradation efficiency of nano-bacteria hybrids was similar to that of wild-type *S. oneidensis*, which is the baseline degradation from anaerobic respiration (biodegradation of less than 5% of dyes after 24 h) [27]. With light irradiation, the degradation efficiency of nano-bacteria hybrids was improved 7-fold. This improvement depends on photoexcitation, suggesting that the semiconductor CdS nanoparticles played a role [28]. For the abiotic sample containing only CdS nanoparticles but no *S. oneidensis*, no significant degradation was observed, even when photoexcited, suggesting that *S. oneidensis* cells provide electrons to fill the holes in the CdS nanoparticle’s valence band to sustain reductive degradation. Taken together, these results suggest a degradation mechanism for the self-assembled nano-bacteria hybrids. The CdS nanoparticles generated excited electrons to reduce dyes under illumination. The paired holes in the valence band received electrons from the anaerobic microbial respiration of *S. oneidensis* to maintain the cycle. This mechanism is consistent with the recognized electron transfer process between *S. oneidensis* and abiotic materials [29,30].

### 3.3. Engineered Living Material with Encapsulated Nano-Bacteria Hybrids

The application of nano-bio hybrids is primarily limited by the fragility and susceptibility of hybrid cells, thus we attempted to transform the as-prepared nano-bacteria hybrids into ELMs to simplify the operation and enhance performance. The ELMs comprised functional live cells and scaffolding matrices, thus integrating the properties (e.g., mechanical properties and porosity) of conventional materials and the distinct functionality of live cells (e.g., responsiveness and regeneration) [31].

**Figure 4:**
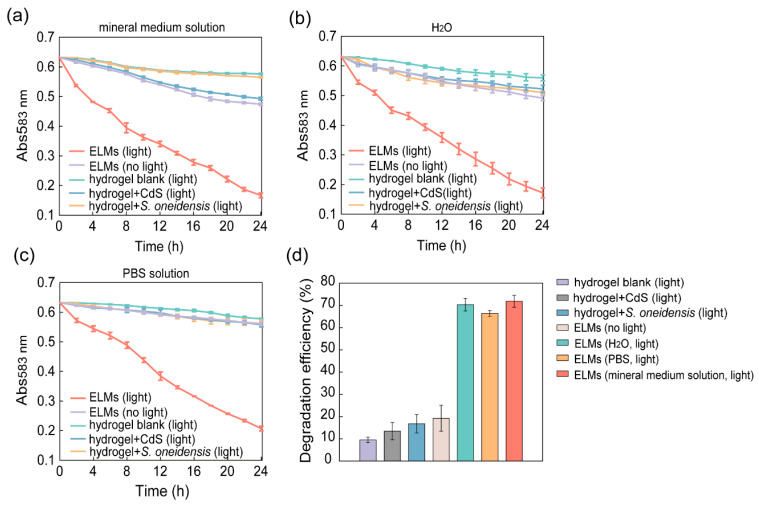
Photocatalytic degradation performance of engineered living materials (ELMs) with encapsulated nano-bacteria hybrids: (**a**) Change in concentration change of trypan blue after treatment with ELMs and controls in mineral medium solution. The ELMs were incubated in a mineral medium solution containing 20 mg/L trypan blue and irradiated by a tungsten filament lamp. Control groups included hydrogels (light), *Shewanella oneidensis* encapsulated in hydrogels (light), CdS encapsulated in hydrogels (light), and ELMs (no light). The photocatalytic degradation performance of ELMs was significantly improved compared to that of wild-type *S. oneidensis* cells encapsulated in hydrogels; (**b**) Change in concentration of trypan blue after treatment with ELMs and controls in water; (**c**) Change in concentration of trypan blue after treatment with ELMs and controls in PBS solution; (**d**) The concentration of trypan blue after 24 h of treatment was compared with the initial concentration to assess the trypan blue degradation efficiency. The concentration was determined by comparing it to a trypan blue calibration curve generated based on Abs_583 nm_. The photocatalytic degradation efficiency of ELMs was not affected by the media (i.e., simulated wastewater, water, and PBS solution). The data are presented as mean ± s.d (*n* = 2).

The self-assembled nano-bacteria hybrids were washed with PBS and resuspended in an LB medium. The suspension was mixed with sodium alginate powder, followed by injection into a CaCl_2_ aqueous solution to form ELM spheres. The photocatalytic degradation performance of trypan blue by ELMs was evaluated (Figure 4a). For comparison with the results for nano-bacteria hybrids not encapsulated, the colony-forming units in the ELMs used were kept consistent with those in Section 3.2. After 24 h of incubation with a mineral medium solution containing trypan blue, ELMs containing nano-bacteria hybrids removed 73.9% of the dye compounds under light irradiation (Figure 4d, Figure S5). Without photoexcitation, only 19.3% of trypan blue could be removed by the ELMs, which is similar to the performance of alginate hydrogels containing *S. oneidensis* cells without engineering (Figure 4d). The slight increase in removal rate compared to the *S. onediensis* cells not encapsulated is attributed to the adsorption of dyes by alginate hydrogels. Taken together, the fabrication process of ELMs does not harm the activity of *S. oneidensis* or the photocatalytic degradation capacity of the nano-bacteria hybrids.

The bioremediation by *S. oneidensis* requires carbon sources to support cell survival [30,32]. As ELMs can contain nutrients, we speculated that photocatalytic degradation by ELMs can be performed in media without carbon sources. The degradation performance of the ELMs was assessed in PBS and water (Figure 4b,c). The ELMs degraded 74.4% and 67.6% of trypan blue in water and PBS solutions, respectively, after 24 h of treatment (Figure 4d, Figure S5). These results demonstrated that ELMs are self-sustaining and can support the survival of nano-bacteria hybrids as well as their ability to biodegrade in solutions without nutrients.

Recent reports have shown that biopolymer scaffolds of ELMs do not interfere with the biosensing and biosynthesis capabilities of encapsulated microbial cells [33,34]. However, there have been few reports examining ELMs containing nano-bacteria hybrids, and little is known about whether biopolymer scaffolds affect the light energy conversion of nano-bacteria hybrids. Our results illustrate the feasibility of fabricating ELMs from nano-bacteria hybrids and demonstrate that nano-bacteria hybrids can continue to perform photocatalytic degradation when encapsulated in biopolymers. Alginate was chosen as the biopolymer scaffold for ELMs because it is a widely used polymer for cell immobilization [35,36,37,38,39]. However, other hydrogel matrices, such as gelatin and bacterial cellulose, can be tested in the future to determine whether nano-bacterial hybrids can survive and perform photocatalytic degradation in other biopolymer scaffolds.

### 3.4. Recycling and Reuse of Encapsulated Nano-Bacteria Hybrids

**Figure 5:**
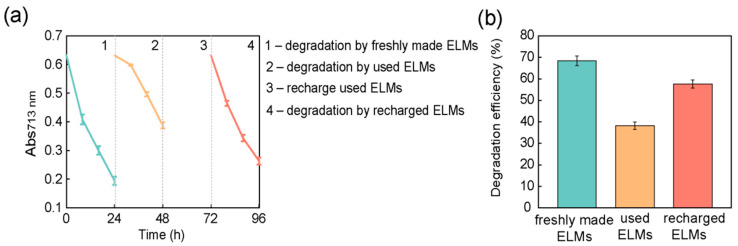
Recycle and recharge engineered living materials (ELMs) after the photocatalytic degradation process: (**a**) Recycling and reuse cycle of ELMs. After degrading trypan blue for 24 h, the ELMs were collected and used to degrade fresh trypan blue solution. The photocatalytic degradation efficiency was lower than that of freshly prepared ELMs. The recycled ELMs were incubated in Luria Bertani (LB) medium solution. After a 24-h incubation, the recharged ELMs were used for the photocatalytic degradation of trypan blue; the observed efficiency was comparable to that of freshly prepared ELMs; (**b**) Degradation efficiency of freshly prepared ELMs, used ELMs, and recharged ELMs after 24 h of treatment. The photocatalytic degradation efficiency of ELMs decreased after incubation with trypan blue but recovered after incubation with LB medium solution. The data are presented as mean ± s.d (*n* = 2).

**Figure 6:**
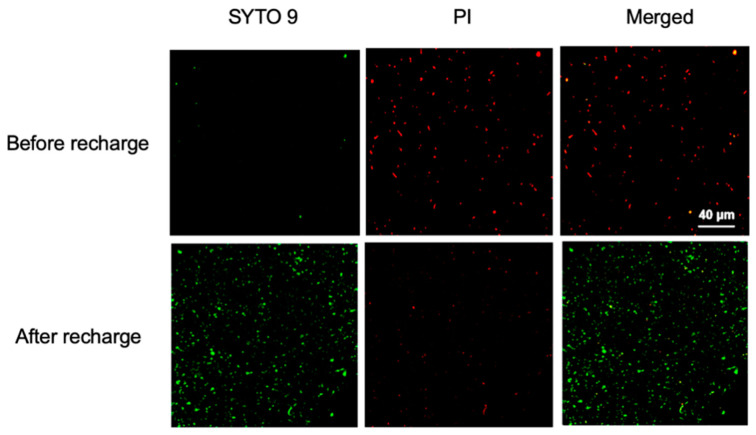
Cell viability in recharged engineered living materials (ELMs). After photocatalytic degradation of trypan blue, spheres were incubated in a culture medium solution. The recharged ELMs spheres were washed with PBS solution and dissolved using EDTA solution. The released cells were co-stained with SYTO 9 and PI and subsequently analyzed under a confocal laser scanning microscope. The majority of the hybrid cells in the recharged ELMs were alive.

The ELMs contain live *S. oneidensis* cells and the nutrients within ELMs support cell survival. The viability of nano-bacteria hybrid cells in ELMs was assessed after degradation treatment. After the degradation of trypan blue, the ELM spheres were crushed and incubated in an LB medium. Colonies formed on LB agar by plating the media confirmed the presence of live cells. Cell identity was confirmed using 16S rDNA sequencing. These results confirmed that there were nano-bacteria hybrid cells that remained alive throughout the treatment process. Because ELMs can preserve the viability of nano-bacteria hybrids, we attempted to recycle and reuse the materials after degradation treatment. After degrading trypan blue, ELM spheres were collected by spin-down and added to fresh trypan blue solution (Figure 5a). The recycled ELMs exhibited photocatalytic degradation capability, but their efficiency was reduced to 40.0% (Figure 5b and Figure S6). The cells were extracted from recycled ELMs to analyze their viability. Live cells were observed in the recycled ELM, but the ratio of live to dead cells decreased to 1:5 (Figure S7). To restore the viability of the encapsulated bacterial cells, the recycled ELM spheres were soaked in fresh LB medium for 24 h to allow cell growth. After incubation, the photocatalytic degradation capacity of trypan blue by ELM spheres was measured again. Our results demonstrate that incubation with LB media can rehabilitate recycled ELMs. The recycled ELMs treated with fresh LB media exhibited a photocatalytic degradation efficiency comparable to that of freshly prepared ELMs (Figure 5b). Incubation with LB media increased the percentage of live *S. oneidensis* cells in ELMs from 18.7% to 67.3% (Figure 6 and Figures S7–S10).

Collectively, these results suggest that the nano-bacteria hybrids can survive in ELMs throughout the degradation treatment, but their viability decreases, probably owing to the toxicity of trypan blue. The recycled ELMs contained fewer live bacterial cells; therefore, their degradation performance was hindered. Recharging used ELMs via incubation with a fresh culture medium could restore their photodegradation capacity by increasing the number of live bacterial cells. Although bacterial cells were observed to have nanoparticles deposited on their surfaces in recharged ELMs (Figure S10), the interaction between the nanoparticles and newly born bacteria needs further investigation.

### 3.5. Safety of Encapsulated Nano-Bacteria Hybrids

Biosafety is always a concern that must be addressed when microorganisms are involved in environmental applications. Although no genetic modification was performed on our nano-bacteria hybrids, the escape risk of the bacterial cells was carefully assessed. The solution was sampled after 12 h or 48 h of degradation and plated on LB agar. No bacterial colonies were observed on the inoculated plates after incubation at 30 °C for more than 24 h, confirming that no detectable leakage of bacterial cells occurred during the degradation process (Figure S11). The ELMs confined the nano-bacteria hybrids and prevented potential biosafety risks associated with live cell escape but allowed the exchange of organic small molecules for degradation.

A commonly overlooked problem in the application of nano-bacteria hybrids is that nanoparticles can peel off from cells. Nanoparticles, especially those containing heavy metals, have nanotoxicity. We observed that by encapsulating nano-bacteria hybrids in ELMs, not only were bacterial cells confined, but the leakage of nanoparticles was also significantly attenuated (Figure S12). The ELM-treated solution contained 15 times less cadmium than the solution treated with nano-bacteria hybrids not encapsulated in hydrogels. Taken together, ELMs can improve the biosafety of applications involving nano-bacteria hybrids and have the potential to reduce the risk of nanotoxicity associated with nanoparticles.

### 3.6. Stability of Encapsulated Nano-Bacteria Hybrids

**Figure 7:**
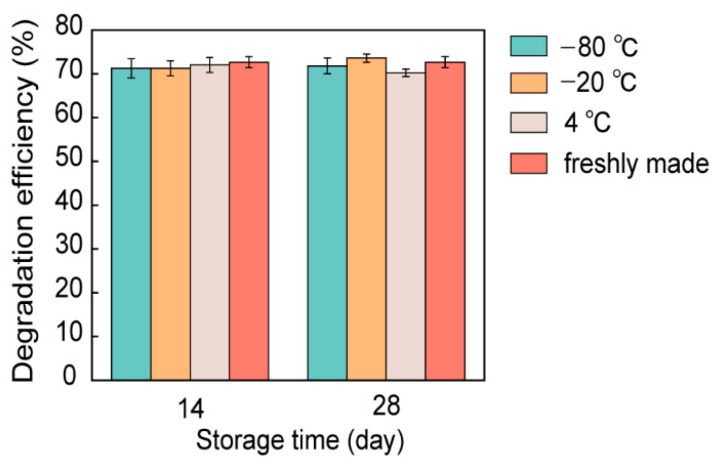
Assessment of the photocatalytic degradation performance of engineered living materials (ELMs) stored under different conditions. The concentration of trypan blue after 24 h of treatment was compared with the initial concentration to assess the trypan blue degradation efficiency. The concentration was determined by comparing it to a trypan blue calibration curve generated based on Abs_583 nm_. The ELMs could retain their photocatalytic degradation capacity for up to 28 days when stored at temperatures lower than 4 °C. The data are presented as mean ± s.d (*n* = 2).

The stability and required storage conditions of a material affect the ease of industrial application. Without additional protection, nano-bacterial hybrids did not survive beyond 3 days. The prepared ELMs contained alginate hydrogels, which are expected to protect nano-bacteria hybrids from environmental stress. To assess the stability of ELMs, ELM spheres containing nano-bacteria hybrids were stored at 4 °C, −20 °C, and −80 °C without additional protection. After 14 and 28 days, the photocatalytic degradation capability of the stored ELM spheres was analyzed. The ELMs stored under different conditions demonstrated similar degradation capacities for trypan blue. The degradation efficiency after storage was comparable to that of freshly prepared ELMs (Figure 7 and Figure S13). These results show that the fabricated ELMs can be stably stored over the long term, with the photocatalytic degradation capacity of nano-bacteria hybrids well preserved.

### 3.7. Regeneration of ELMs

**Figure 8:**
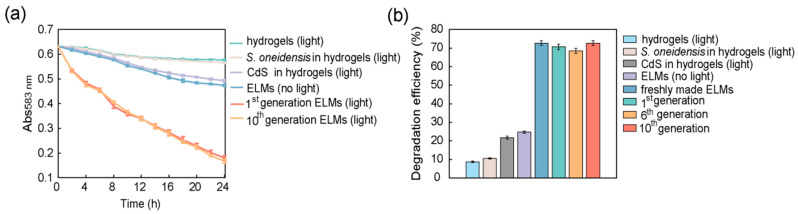
Assessment of the regenerative properties of engineered living materials (ELMs): (**a**) Change in concentration of trypan blue after treatment with the 1st and 10th generation of regenerated ELMs. Live cells were extracted from recycled ELMs and used to regenerate bacterial culture for the biosynthesis of nano-bacteria hybrids. The as-prepared nano-bacteria hybrids were encapsulated in alginate hydrogels to regenerate ELMs. The regeneration process was repeated for 10 generations; (**b**) The concentration of trypan blue after 24 h of treatment was compared with the initial concentration to assess the trypan blue degradation efficiency. The concentration was determined by comparing it to a trypan blue calibration curve generated based on Abs_583 nm_. Regenerated ELMs exhibited photocatalytic degradation efficiency comparable to that of freshly prepared ELMs. No significant difference was observed between the degradation performance of different generations of ELMs. The data are presented as mean ± s.d (*n* = 2).

There has been growing interest in the development of regenerative ELMs [40,41,42]. The observation that nano-bacteria hybrids remain alive in ELMs suggests that recycled ELMs can be utilized to regenerate successive generations of ELMs. To assess the regenerative potential of the ELMs containing nano-bacteria hybrids, the spheres were crushed and incubated in LB medium solution after degradation treatment. Such a solution containing live bacterial cells was used to regenerate bacterial cultures for the biosynthesis of nano-bacteria hybrids. The as-prepared nano-bacteria hybrids were encapsulated in alginate hydrogels to regenerate ELMs. The regeneration process was repeated for 10 generations. For each generation of ELMs, the photocatalytic degradation capacity for trypan blue was measured and compared with that of the parental generation, which showed no significant decline (Figure 8 and Figure S14). The nano-bacteria hybrids in our ELMs could be extracted and used to regenerate more ELMs. The regenerated ELMs exhibited photocatalytic degradation performance that was comparable to that of the parental generation, suggesting that nano-bacteria hybrids in ELMs did not experience degeneration. These results highlight the sustainability of the proposed materials. The regenerative property makes it practical to construct ELMs in the field, which is useful when the ELMs are damaged or shipment of large amounts of ELMs is impractical.

### 3.8. Degradation of Other Dyes by Nano-Bacteria Hybrids and ELMs

Real wastewater has a complex composition; thus, techniques that can be applied to various contaminants are appealing. The ELM with nano-bacteria hybrids would be practically more useful if it could be applied to the reductive degradation of other types of contaminants as well. Because *S. oneidensis* is capable of biodegrading various organic pollutants other than azo dyes, such as sulfonamide and nitroso compounds [43,44], we tested the degradation capacity of ELMs for NGB. Naphthol green B contains nitroso groups, which can be converted into amino groups by reductive degradation. The degradation efficiency of NGB by *S. oneidensis*-CdS nano-bacteria hybrids and ELMs was assessed. The degradation of NGB led to a decrease in the absorption at 713 nm, which was used to measure its concentration. Treatment of 100 mg/L NGB solution with nano-bacteria hybrids for 24 h under light irradiation consumed more than 60% of the contaminants, which was a marked improvement compared to that with only *S. oneidensis* cells or the no-light control group (Figure S15). After encapsulating nano-bacteria hybrids in alginate hydrogels, the photocatalytic degradation efficiency of NGB by ELMs was not affected (Figure S15). The degradation efficiency of NGB by the ELMs was not hindered after long-term storage (Figure S16). The degradation performance of regenerated ELMs toward NGB was also evaluated and was found to be comparable to that of freshly prepared ELMs (Figure S17). These results demonstrate that ELMs constructed from *S. oneidensis*-CdS nano-bacteria hybrids have the potential to biodegrade recalcitrant chemicals with various functional groups.

## 4. Conclusions

We developed a method to self-assemble *S. oneidensis* with bacterially biosynthesized CdS nanoparticles under pseudo anaerobic conditions. The self-assembled nano-bacteria hybrids can be directly applied to degradation without the need to isolate and purify the nanoparticles. To improve the resistance of the material to environmental stress, nano-bacteria hybrids were encapsulated in ELMs using an alginate matrix. Our results suggest that immobilization with alginate hydrogels preserves the photocatalytic properties of nano-bacteria hybrids. More importantly, ELMs could enhance the viability of nano-bacteria hybrids. Outside ELMs, nano-bacteria hybrids cannot survive beyond 3 days, whereas inside ELMs, their viability and photocatalytic degradation capacity remained unhindered after 4 weeks.

The unique properties of ELMs in this study (i.e., recyclable, stable, and regenerative) make nano-bacteria hybrids a step closer to becoming wastewater treatment materials. To further explore these possibilities, the degradation performance and stability of the ELMs will be examined in real wastewater containing diverse chemicals in future investigation.

## Data Availability

The authors confirm that the data supporting the findings of this study are available within the article and its supplementary materials.

## Supplementary Materials

The following supporting information can be downloaded at: https://www.mdpi.com/article/10.3390/microorganisms10122501/s1, Figure S1: The color change of the solution after biosynthesis of CdS; Figure S2: EDS analysis of the CdS nanoparticles isolated from nano-bacteria hybrids; Figure S3: Fluorescence spectrum of the CdS nanoparticles isolated from nano-bacteria hybrids; Figure S4: Photocatalytic degradation efficiency of nano-bacteria hybrids towards trypan blue; Figure S5: Photocatalytic degradation performance of ELMs with encapsulated nano-bacteria hybrids in various media; Figure S6: The degradation efficiency of ELMs during the recycling and recharging process was monitored; Figure S7: Flow cytometry analysis of cells in ELMs before recharge; Figure S8: Flow cytometry analysis of cells in ELMs after recharge; Figure S9: Cell viability in ELMs assessed by plate counting before and after recharge; Figure S10: TEM image of nano-bacteria hybrids extracted from ELMs; Figure S11: Biosafety assessment of ELMs; Figure S12: Assessment of nanoparticle leakage from nano-bacteria hybrids and ELMs; Figure S13: Photocatalytic degradation performance of trypan blue by ELMs stored under different conditions; Figure S14: Photocatalytic degradation performance of trypan blue by regenerated ELMs; Figure S15: Photocatalytic degradation of naphthol green B by nano-bacteria hybrids and ELMs; Figure S16: Photocatalytic degradation performance of NGB by ELMs stored under different conditions; Figure S17: Photocatalytic degradation performance of NGB by regenerated ELMs.
