# Supplementary material for: Living and Regenerative Material Encapsulating Self-Assembled Shewanella oneidensis-CdS Hybrids for Photocatalytic Biodegradation of Organic Dyes
Source: Microorganisms. 2022 Dec 16;10(12):2501. doi: 10.3390/microorganisms10122501 (PMC9781410; doi:10.3390/microorganisms10122501)
Supplement: Supplementary file 1 [file microorganisms-10-02501-s001.zip › microorganisms-2064991-supplementary.pdf]

Supplementary Information

Living and Regenerative Material Encapsulating Self-Assembled *Shewanella oneidensis*-CdS Hybrids for Photocatalytic Biodegradation of Organic Dyes

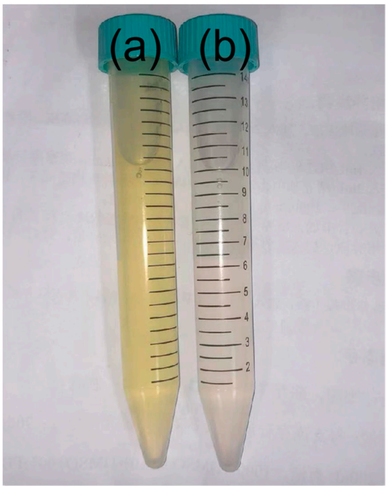

Figure S1. The color of the solution changed to yellow after biosynthesis of CdS.(a) with $CdCl_2$ addition, (b) without $CdCl_2$ addition.

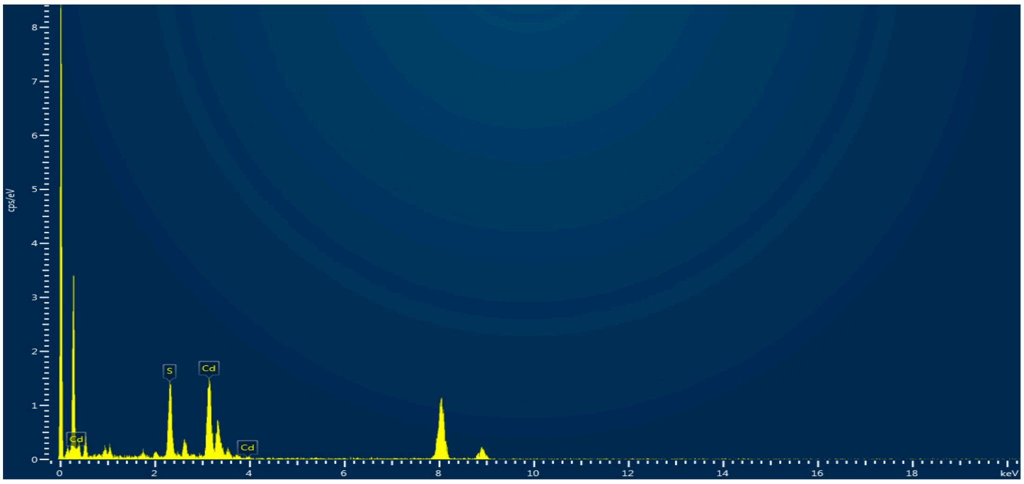

Figure S2. EDS analysis of the CdS nanoparticles isolated from nano-bacteria hybrids. The biosynthesized nanoparticles consist of Cd and S elements with a ratio of 1:1 (Cd: 53%, S: 46%).

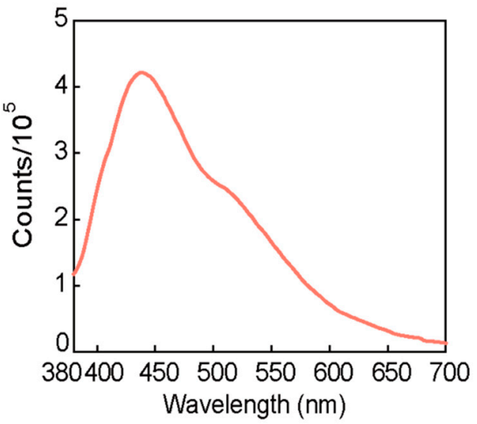

Figure S3. Fluorescence spectrum of the CdS nanoparticles isolated from nano-bacteria hybrids. The CdS nanoparticles were suspended in a mineral solution and excited at 350 nm. The fluorescence spectrum exhibited an emission peak at 450 nm, which was comparable with that of chemically synthesized CdS.

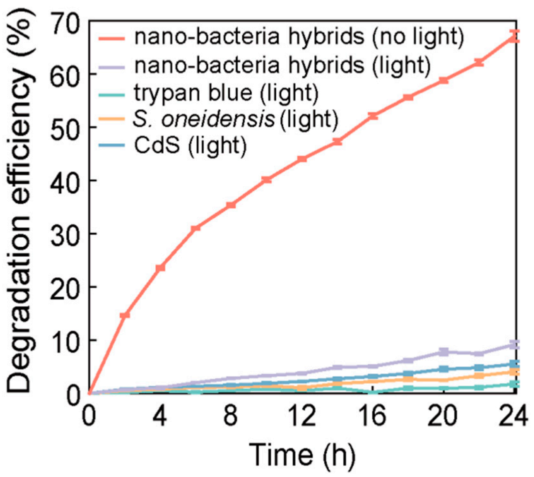

Figure S4. Photocatalytic degradation efficiency of nano-bacteria hybrids towards trypan blue. The concentration of trypan blue over the treatment process was compared with the initial concentration to assess the trypan blue degradation efficiency. The concentration was determined according to a trypan blue calibration curve generated based on $Abs_{583\ nm}$. The data are presented as mean ± s.d (n=2).

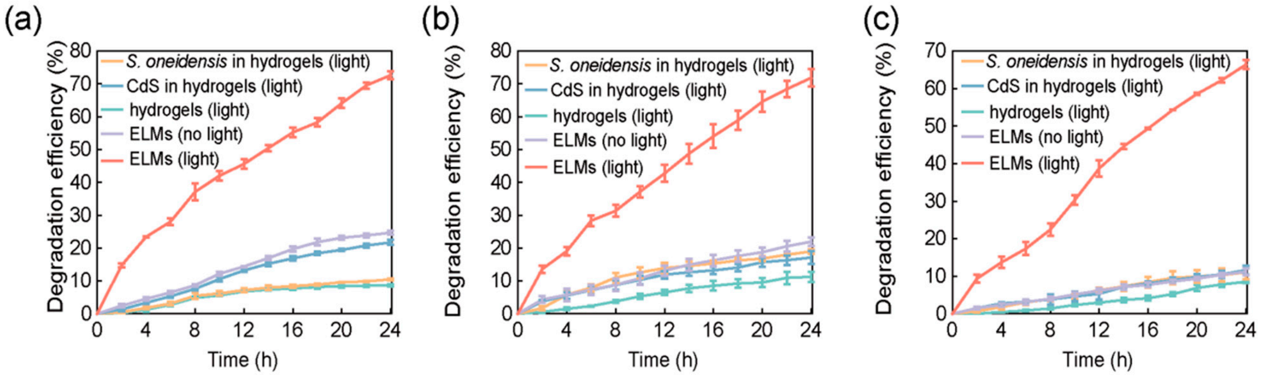

Figure S5. Photocatalytic degradation performance of ELMs with encapsulated nano-bacteria hybrids in various media, (a) mineral medium solution, (b) water, (c) PBS solution.The data are presented as mean ± s.d (n=2).

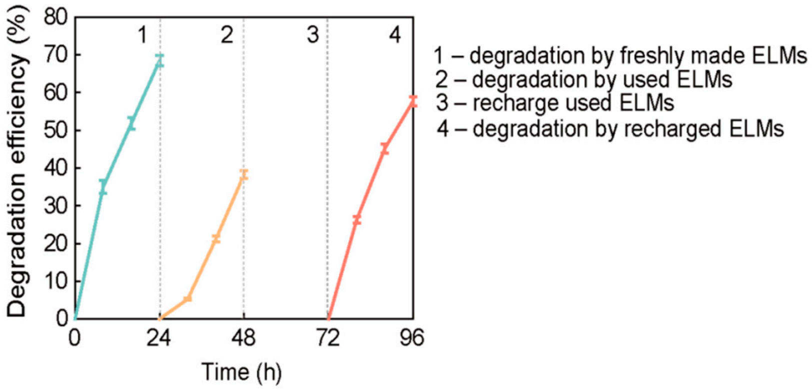

Figure S6. The degradation efficiency of ELMs during the recycling and recharging process was monitored. The photocatalytic degradation efficiency of ELMs decreased after incubation with trypan blue, but was recovered after incubation with LB medium solution. The data are presented as mean ± s.d (n=2).

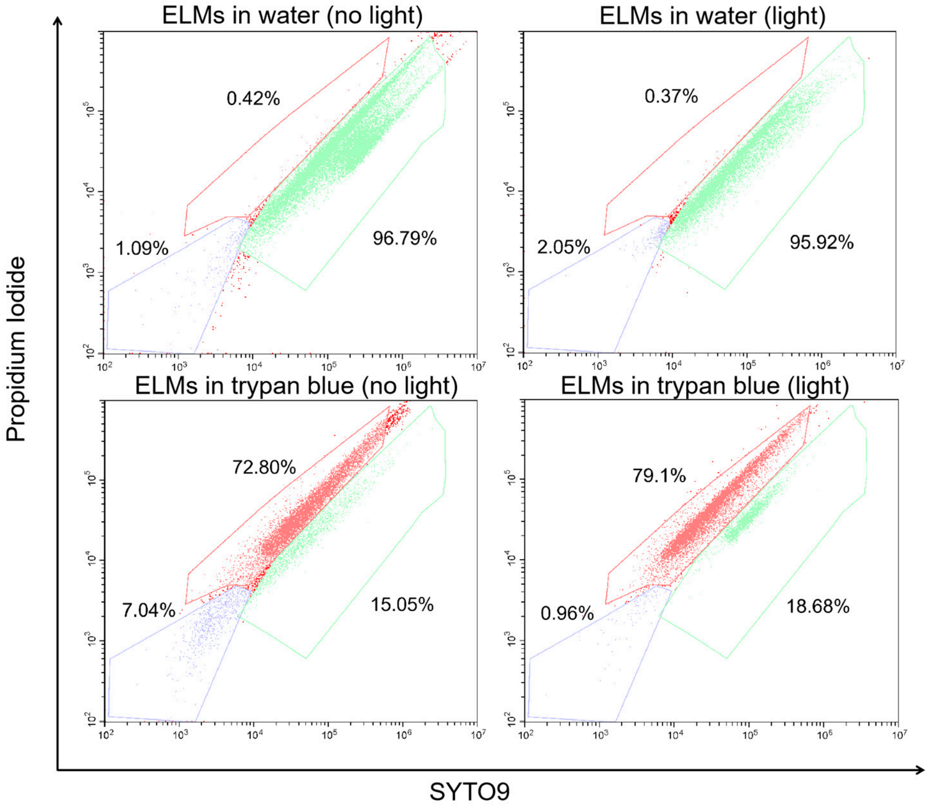

Figure S7. Flow cytometry analysis of cells in ELMs before recharge. After 24-hr incubation in trypan blue solution (with or without irradiation), ELMs were collected and dissolved using EDTA solution. Samples were co-stained with SYTO9 and PI dyes for 25 min and then analyzed on a flow cytometer. ELMs incubated in water were used as controls. Live cells represented only 15-20% of the total cells, compared to approximately 95% in the controls.

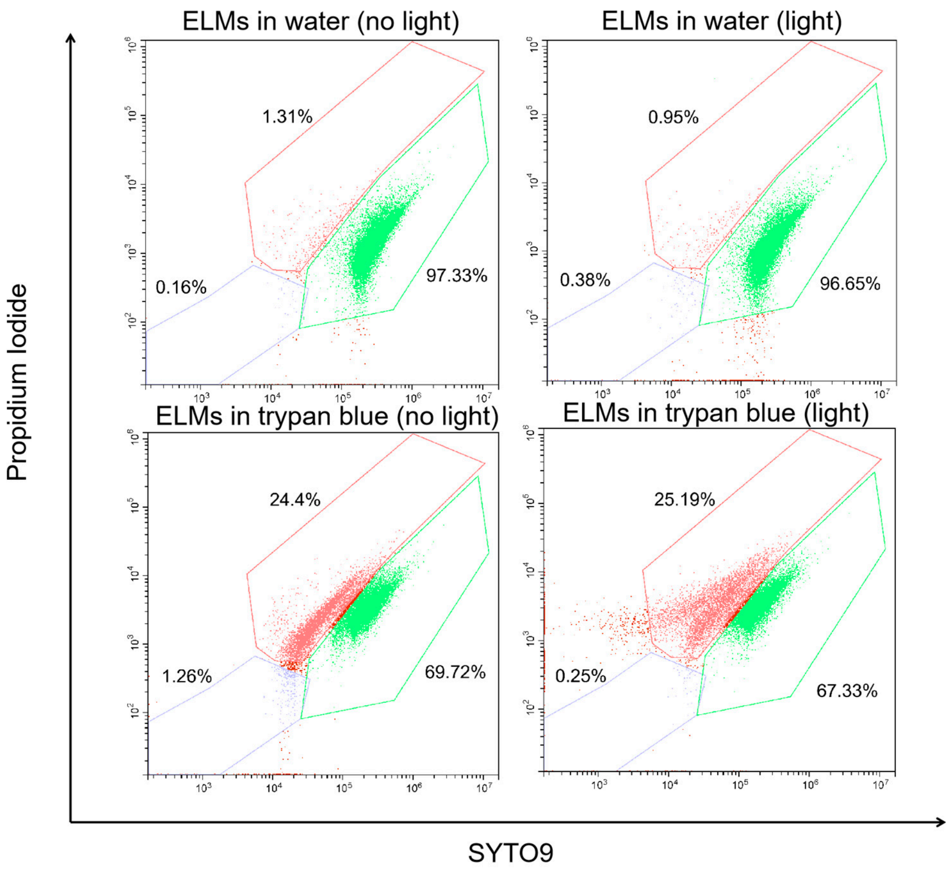

Figure S8. Flow cytometry analysis of cells in ELMs after recharge. After 24-hr incubation in trypan blue solution, ELMs were incubated in LB medium at 30 °C/250 rpm. After another 24 hr, ELMs were collected and dissolved using EDTA solution. The samples were co-stained with SYTO9 and PI dyes for 25 min and then analyzed on a flow cytometer. The percentage of live cells within the ELMs increased to approximately 70% of the total cells.

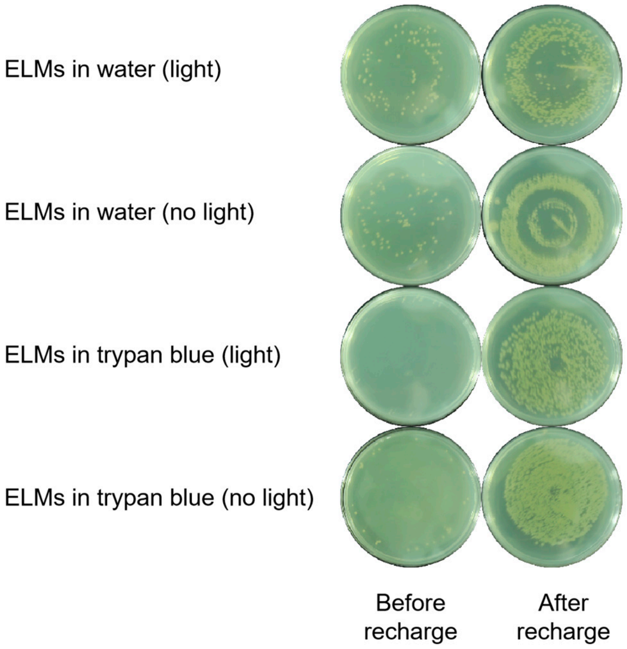

Figure S9. Cell viability in ELMs assessed by plate counting before and after recharge. Before and after recharge, ELMs spheres were collected and washed with PBS solution and dissolved using EDTA solution. A dilution from the resulting solution was plated on an LB agar plate and incubated at 30 °C for 24 hr. There were much fewer viable cells in ELMs treated with trypan blue compared to ELMs incubated in water. The number of live cells in ELMs was restored after incubating in LB culture media.

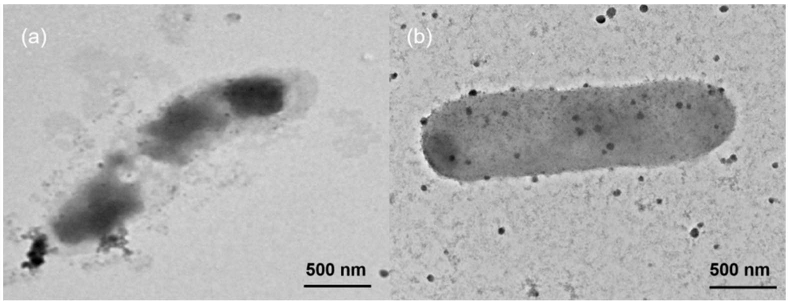

Figure S10. TEM image of nano-bacteria hybrids extracted from ELMs. (a) After degrading trypan blue, ELMs spheres were dissolved by EDTA solution. The nano-bacteria hybrids were subjected to TEM analysis. Lysis of bacterial cells was observed. (b) The used ELMs were incubated in LB medium solution to be recharged. TEM images of nano-bacteria hybrids extracted from the recycled ELMs showed CdS deposited on intact bacteral cells.

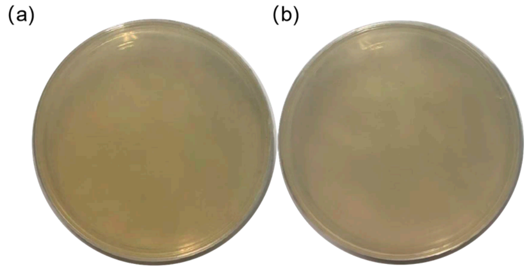

Figure S11. Biosafety assessment of ELMs. After 12-hr (a) and 36-hr (b) degradation of trypan blue solution by ELMs, the ELMs were removed by filtration. The residual solution was plated on an LB agar plate and incubated at 30 °C. After more than 24 hours, no colonies formed on the plates, indicating that no cells had escaped from ELMs.

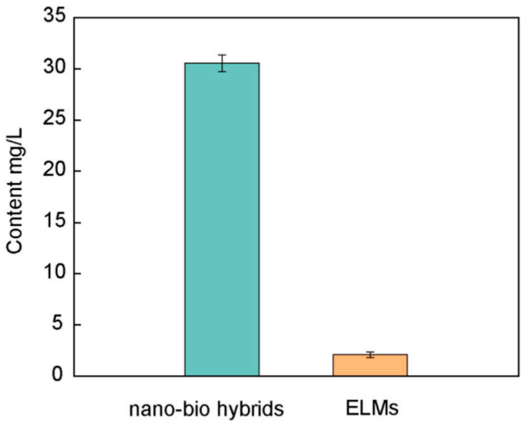

Figure S12. Assessment of nanoparticle leakage from nano-bacteria hybrids and ELMs. After 24 hr of photocatalytic degradation by ELMs or nano-bacteria hybrids, 5 mL of the solution was collected and treated with acid digestion. The digested solution was diluted with water and analyzed on Aglient 5110 ICP-OES to quantify the cadmium contents. The cadmium content in the ELMs-treated solution was 15 times lower than that in the solution treated with nano-bacteria hybrids not encapsulated in hydrogels, suggesting that ELMs significantly attenuated the leakage of nanoparticles. The data are presented as mean ± s.d (n=4).

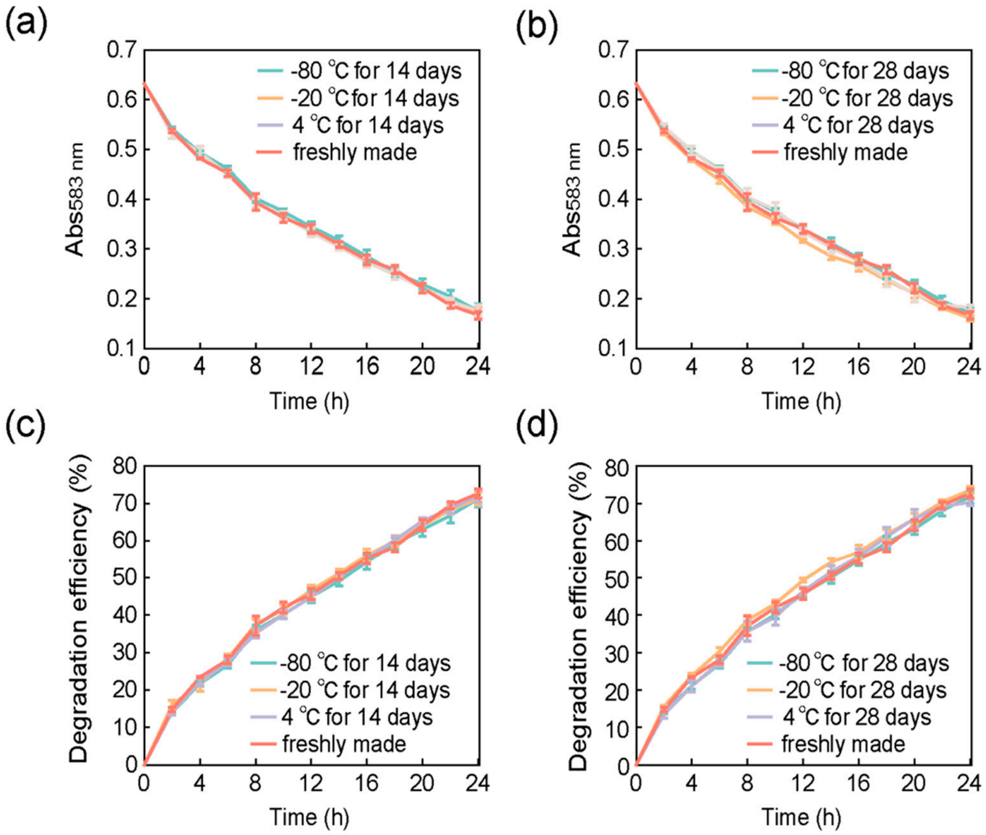

Figure S13. Photocatalytic degradation performance of trypan blue by ELMs stored under different conditions. (a) Concentration change of trypan blue after treatment with ELMs stored at 4 °C/-20 °C/-80 °C for 14 days. No significant difference was observed between the photocatalytic degradation performance of stored ELMs and that of freshly made ELMs. (b) Concentration change of trypan blue after treatment with ELMs that were stored at 4 °C/-20 °C/-80 °C for 28 days. No significant difference was observed between the photocatalytic degradation performance of stored ELMs and that of freshly made ELMs. Photocatalytic degradation performance of trypan blue by ELMs stored under different conditions. ELMs were stored at 4 °C, -20 °C, and -80 °C for 14 days (c) and 28 days (d). No significant differences were observed between the photocatalytic degradation performance of stored ELMs and freshly made ELMs. The data are presented as mean ± s.d (n=2).

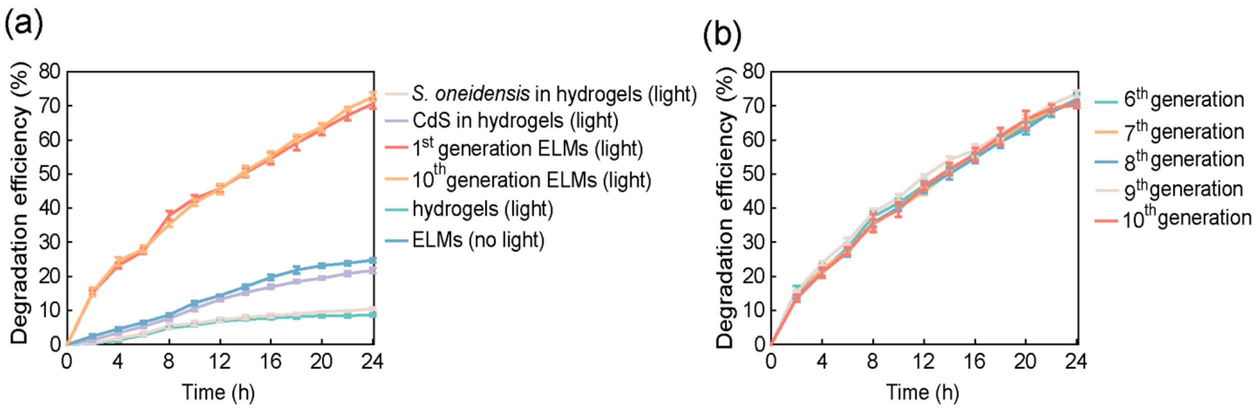

Figure S14. Photocatalytic degradation performance of trypan blue by regenerated ELMs. (a) The 1st and 10th generation of regenerated ELMs exhibited degradation efficiency towards trypan blue comparable to that of freshly made ELMs. (b) The degradation efficiency of different generations of regenerated ELMs was compared. No significant differences were observed between different generations. The data are presented as mean ± s.d (n=2).

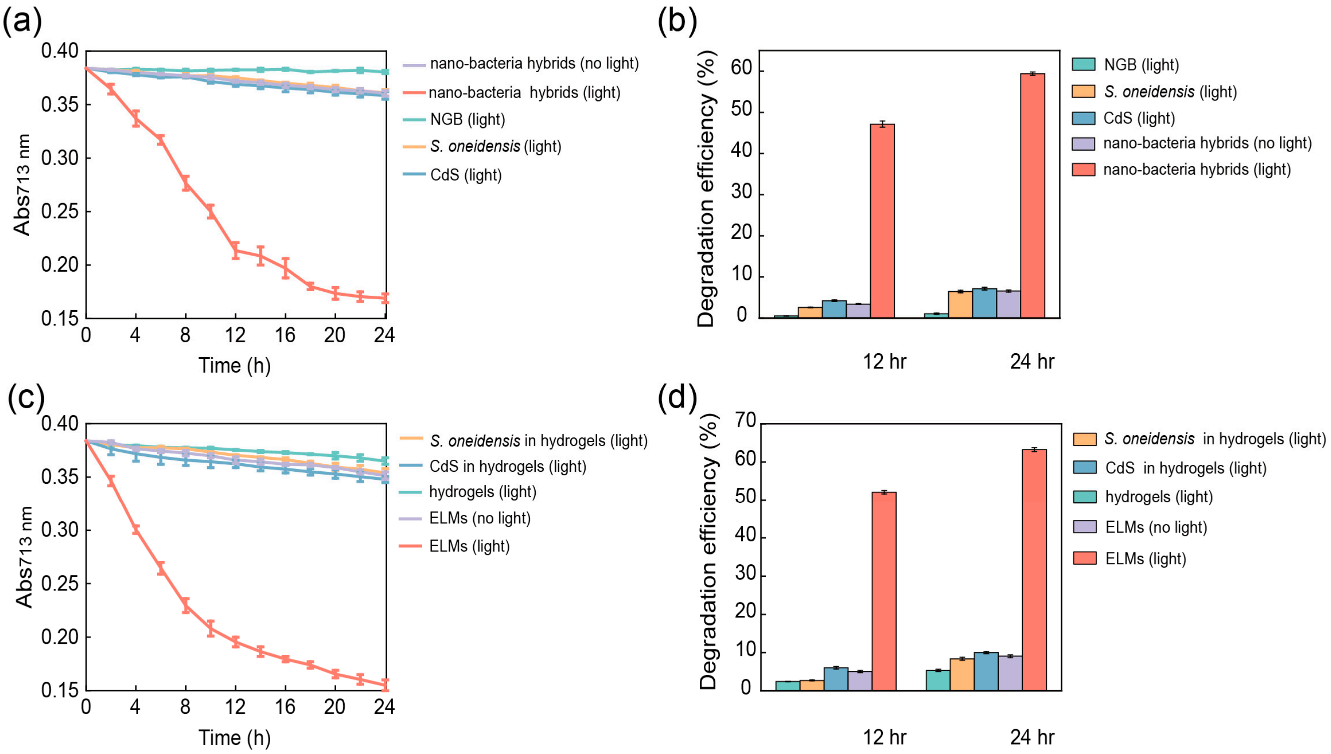

Figure S15. Photocatalytic degradation of naphthol green B by nano-bacteria hybrids and ELMs. (a) Concentration change of NGB after treatment with nano-bacteria hybrids and controls. Nano-bacteria hybrids were incubated in mineral medium solution containing 100 mg/L NGB and irradiated by a tungsten filament lamp. The absorbance of the reaction mixture at 713 nm was used to determine the concentration change of NGB. Control groups included NGB only (with light), *S. oneidensis* only (with light), CdS only (with light), and nano-bacteria hybrids (without light). The photocatalytic degradation performance of nano-bacteria hybrids was significantly improved compared to that of wild-type *S. oneidensis* cells. (b) The concentration of NGB after 12/24 hr of treatment with nano-bacteria hybrids was compared to the initial concentration to assess the NGB degradation efficiency. The concentration was determined according to a NGB calibration curve generated based on $Abs_{713\ nm}$. The photocatalytic degradation efficiency of nano-bacteria hybrids towards NGB was 10 times better than that of wild-type *S. oneidensis* cells. (c) Concentration change of NGB after treatment with ELMs and controls. ELMs were incubated in mineral medium solution containing 100 mg/L NGB, and irradiated by a tungsten filament lamp. Control groups included hydrogels (light), *S. oneidensis* encapsulated in hydrogels (light), CdS encapsulated in hydrogels (light), and ELMs (no light). The photocatalytic degradation performance of ELMs was considerably improved compared to that of wild-type *S. oneidensis* cells encapsulated in hydrogels. (d) The photocatalytic degradation efficiency of ELMs was 8 times better than that of wild-type *S. oneidensis* cells encapsulated in ELMs. The data are presented as mean ± s.d (n=2).

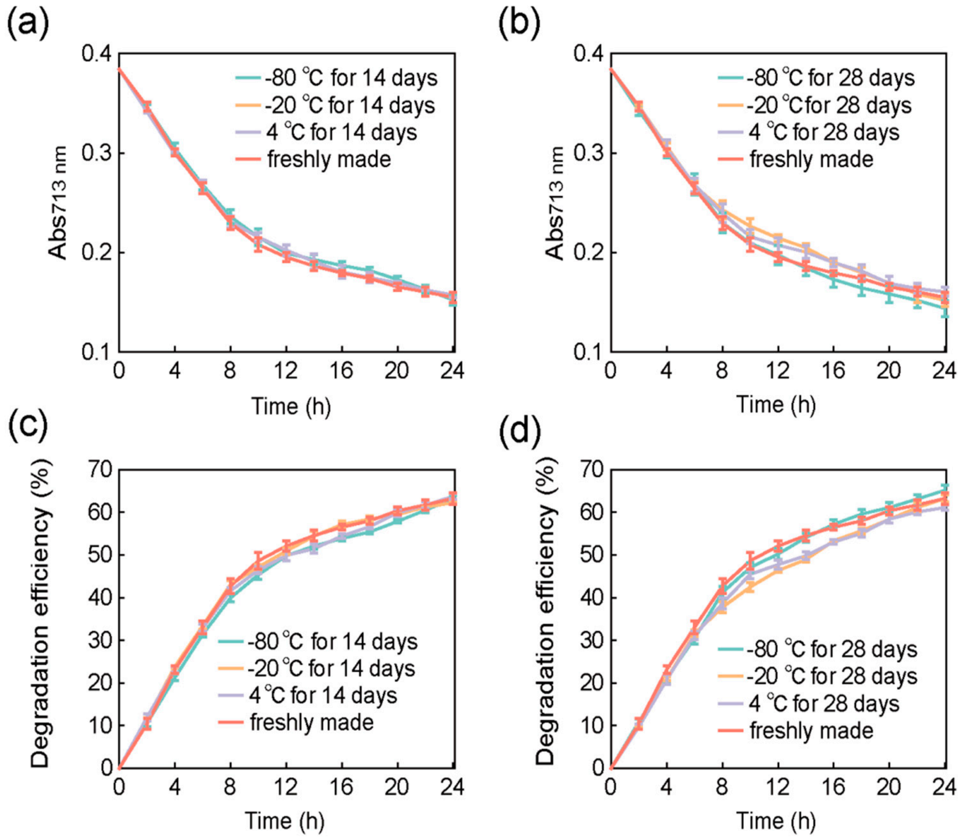

Figure S16. Photocatalytic degradation performance of NGB by ELMs stored under different conditions. ELMs were stored at 4 °C, -20 °C, and -80 °C for 14 days (a/c) and 28 days (b/d). No significant differences were observed between the photocatalytic degradation performance of stored ELMs and freshly made ELMs. The data are presented as mean ± s.d (n=2).

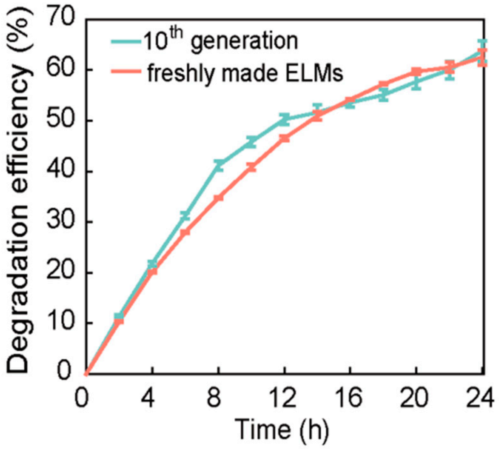

Figure S17. Photocatalytic degradation performance of NGB by regenerated ELMs. No significant differences were observed between the 10th generation of regenerated ELMs and the freshly made ELMs. The data are presented as mean ± s.d (n=2).
